# Preparation of Porous Poly(Styrene-Divinylbenzene) Microspheres and Their Modification with Diazoresin for Mix-Mode HPLC Separations

**DOI:** 10.3390/ma10040440

**Published:** 2017-04-22

**Authors:** Bing Yu, Tao Xu, Hailin Cong, Qiaohong Peng, Muhammad Usman

**Affiliations:** 1 Institute of Biomedical Materials and Engineering, College of Chemistry and Chemical Engineering, Qingdao University, Qingdao 266071, China; yubingqdu@yahoo.com (B.Y.); amnano@163.com (T.X.); weljoe@163.com (Q.P.); usman.latif21@gmail.com (M.U.); 2 Laboratory for New Fiber Materials and Modern Textile, Growing Base for State Key Laboratory, College of Materials Science and Engineering, Qingdao University, Qingdao 266071, China

**Keywords:** polystyrene, seeded polymerization, macroporous structures, diazoresin, high performance liquid chromatography

## Abstract

By using the two-step activated swelling method, monodisperse porous poly(styrene-divinylbenzene) (P(S-DVB)) microparticles were successfully synthesized. The influence of porogens, swelling temperatures and crosslinking agents on the porosity of porous microparticles was carefully investigated. Porous P(S-DVB) microparticles were used as a packing material for high performance liquid chromatography (HPLC). Several benzene analogues were effectively separated in a stainless-steel column as short as 75 mm due to the high specific surface area of the porous microparticles. Porous P(S-DVB) microparticles were further sulfonated and subsequently modified with diazoresin (DR) via electrostatic self-assembly and UV (ultraviolet) radiation. After treatment with UV light, the ionic bonding between sulfonated P(S-DVB) and DR was converted into covalent bonding through a unique photochemistry reaction of DR. Depending on the chemical structure of DR and mobile phase composition, the DR-modified P(S-DVB) stationary phase performed different separation mechanisms, including reversed phase (RP) and hydrophilic interactions. Therefore, baseline separations of benzene analogues and organic acids were achieved by using the DR-modified P(S-DVB) particles as packing materials in HPLC. According to the π–π interactional difference between carbon rings of fullerenes and benzene rings of DR, C_60_ and C_70_ were also well separated in the HPLC column packed with DR-modified P(S-DVB) particles.

## 1. Introduction

In the last few decades, a lot of investigation has been done on the synthesis and properties of porous P(S-DVB) beads. The activated swelling method, developed by Ugelstad et al. was utilized for the synthesis of monodisperse porous particles in the range of 1–20 μm [1,2,3]. Recently, a lot of research has been conducted to study the different properties of monodisperse polystyrene microspheres with porous structure [4,5,6,7,8,9,10,11], since they have high specific surface area, tunable pore morphology, mechanical stability and strong adsorption [12,13,14]. The widely-used techniques for the synthesis of porous P(S-DVB) beads include activated swelling [15], seeded emulsion polymerization [16], precipitation polymerization [17], template imprinting [18,19] and membrane techniques [20,21].

Due to lower back pressure, a more regular flow regime in the column and higher resolutions, which result from their excellent permeability and high surface area, the monodisperse microspheres with porous structures have been widely used in chromatographic columns as packing materials [22,23,24,25,26,27]. For example, Unsal et al. [28] investigated the effect of the monomer/seed latex ratio for the chromatographic performance of monodisperse porous particles produced by a modified seeded polymerization method. Fréchet et al. [29,30,31,32] explored the impact of different surface chemistries of monodisperse macroporous particles on the chromatographic behavior.

In this paper, we synthesized monodisperse porous P(S-DVB) microspheres by utilizing the activated swelling technique and investigated the effect of the porogen ratio, temperature and crosslinking agents. The porous P(S-DVB) microparticles were further sulfonated with concentrated sulfuric acid and subsequently modified with diazoresin(DR)via electrostatic self-assembly and UV (ultraviolet) radiation. These porous microspheres were applied as column packing material in mix-mode HPLC analysis of benzene analogs, organic acids and fullerenes.

## 2. Results and Discussion

### 2.1. Synthesis of Porous P(S-DVB) Microparticles

**Figure 1:**
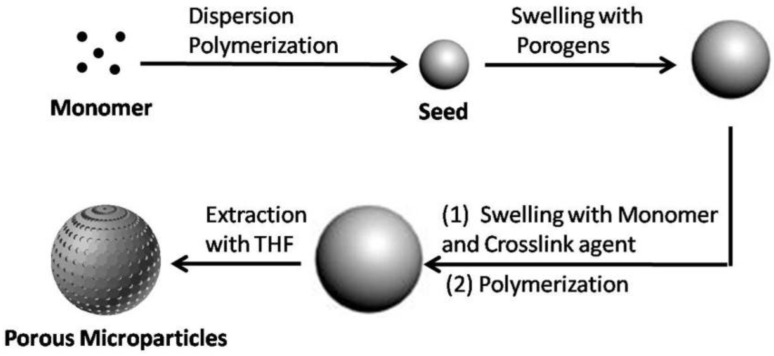
Schematic illustration of the preparation of porous polymer microparticles.

**Figure 2:**
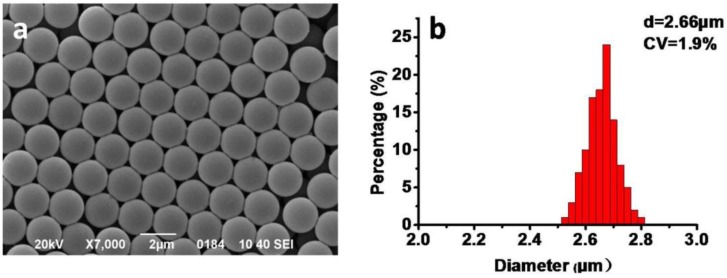
SEM images of 2.7 µm PS seed microspheres synthesized by dispersion polymerization (**a**) and the histogram of the distribution of PS seed microspheres (**b**).

As illustrated in Figure 1, the synthesis process of monodisperse porous polymer microparticles was based on monodisperse linear polystyrene(PS) seed particles, undergoing porogen swelling, monomer and crosslinker swelling, polymerization and extraction. Monodisperse PS seed particles prepared by dispersion polymerization have an average diameter of 2.7 µm, and the coefficient of deviation (CV) is only about 1.9% (Figure 2). The monodispersity was inherited when porous polymer microparticles were prepared. Dibutyl phthalate (DBP) is a seed swelling activator and porogen is a classical plasticizer. Toluene as a porogen is an excellent solvent of polystyrene. These two organic solvents can be diffused into PS seed particles from their emulsion. After being swollen with porogen, monomer, crosslinker and initiator, polymerization occurs in the seed particles to form an interpenetrating polymer network. Linear PS and porogens stay in the crosslinked polymer network and are then extracted by tetrahydrofuran (THF) to generate the porous structure. The preparation conditions of porous polymer microparticles are summarized in Table 1.

**Figure 3:**
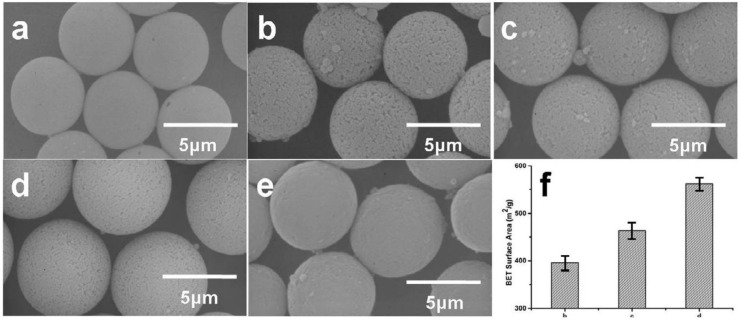
SEM images of porous P(S-DVB) microparticles synthesized by using different volume ratios of toluene to DBP: (**a**) 0:2.4; (**b**) 0.6:1.8; (**c**) 1.2:1.2; (**d**) 1.8:0.6; (**e**) 2.4:0; (**f**) the BET surface areas of b, c and d.

The porosity of porous polymer microparticles is greatly influenced by porogens. Figure 3 shows the morphologies of porous polymer microparticles prepared by using different ratios of toluene to DBP as porogens. When only DBP was applied, no pores were observed on the surface of the microparticles (Figure 3a). When the toluene ratio was increased, different sizes of pores with uniform distribution appeared on the surface of the microparticles (Figure 3b–d). When only toluene was used, a few pores were observed (Figure 3e). The BET (the abbreviation of Brunauer, Emmett and Teller) (Figure 3f) result showed that the largest surface area was obtained when the toluene to DBP ratio was 3:1.

**Figure 4:**
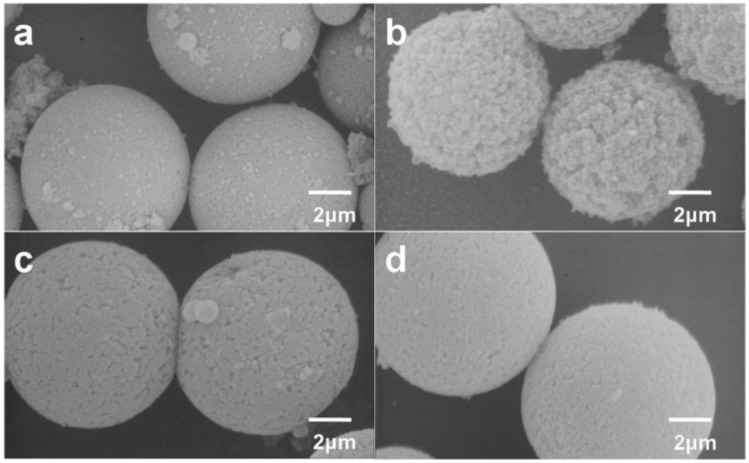
SEM images of the porous P(S-DVB) microparticles synthesized by applying different swelling temperatures: (**a**) 15 °C; (**b**) 25 °C; (**c**) 35 °C; (**d**) 45 °C.

The swelling temperature can also influence the morphology of porous P(S-DVB) microparticles. As shown in Figure 4, porous P(S-DVB) microparticles have the roughest surface when 25 °C of swelling temperature is applied. A smoother surface and smaller pore size were generated by either decreasing or increasing the swelling temperature. Temperature generally has a great impact on the swelling kinetics. PS seeds might not be well swelled by porogens and monomers under low temperature, leading to dense porous structures. On the contrary, high temperature would accelerate prepolymerization of monomers during swelling, leading to a smoother surface and smaller pore size.

**Figure 5:**
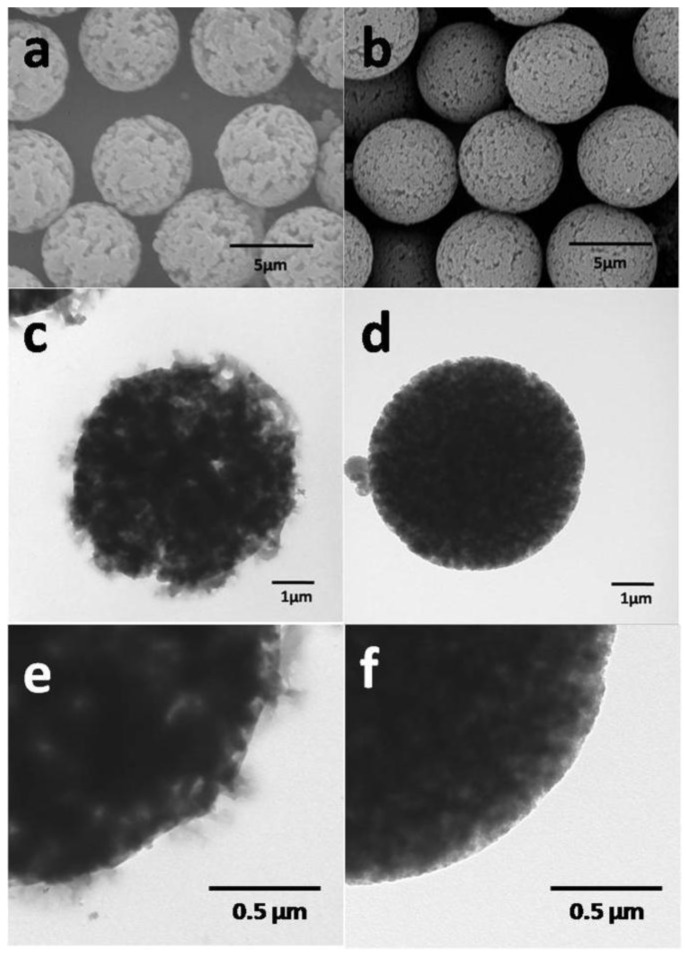
SEM and TEM images of porous particles prepared by using different crosslinking agents: (**a**,**c**,**e**) EDMA; (**b**,**d**,**f**) DVB.

Using different crosslinking agents for synthesizing porous P(S-DVB) microparticles presents different morphologies. Figure 5 shows the SEM and TEM images of porous particles that were synthesized by using different crosslinking agents. We can see that the porous microparticles have larger pore size when ethylene dimethacrylate (EDMA) is used as a crosslinker, while the microparticles have more uniform pore distribution when DVB is used as a crosslinker. The miscibility and phase separation difference of PS with EDMA and DVB may be the main reason behind this phenomenon.

### 2.2. Porosity

**Figure 6:**
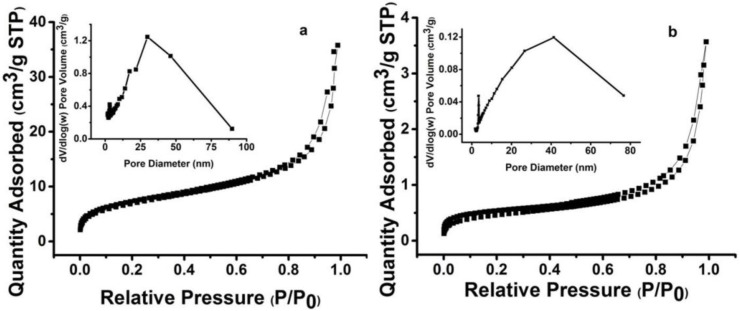
Nitrogen adsorption–desorption isotherms of P(S-DVB)-3 (**a**) and P(S-EDMA) (**b**). Insets are the distributions of pore size.

The surface areas are calculated from N_2_ sorption isotherms by utilizing the BET method (Figure 6). The specific surface area of the porous P(S-DVB)-3 and P(S-EDMA) microparticles are 464 m^2^/g and 37 m^2^/g, respectively, which shows that the crosslinkers have a great impact on the porosity of porous polymer particles. The highest specific surface area can reach 562 m^2^/g of porous P(S-DVB)-4 microparticles calculated by using the BET method. The average pore size of the P(S-DVB)-3 and P(S-EDMA) are 10 nm and 18 nm, respectively. The hysteresis loops at moderate to high relative pressure, as shown in Figure 6, indicate mesoporous structures of porous P(S-DVB)-3 and P(S-EDMA) microparticles.

### 2.3. DR-Modified P(S-DVB) Microparticles

**Figure 7:**
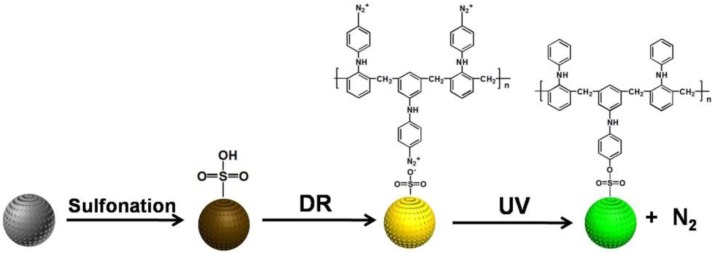
Schematic illustration of the diazoresin (DR) modification of P(S-DVB).

DR-modified porous P(S-DVB) microparticles were synthesized by following the routes in Figure 7. The porous P(S-DVB) microparticles were first sulfonated by concentrated sulfuric acid to form negatively charged porous microparticles, and the positively charged DR was coated onto the surface of porous microparticles via electrostatic interaction. The electrostatic interaction was completely transformed into stable covalent bonds after UV light treatment. The DR was first converted into its phenyl cationic form after releasing N_2_ upon UV irradiation, then a S_N_ 1 type of nuclear displacement by sulfonate occurs [33]. The specific surface areas of DR-modified porous P(S-DVB) microparticles (396 m^2^/g) and the distribution of pore size are shown in Figure S1.

**Figure 8:**
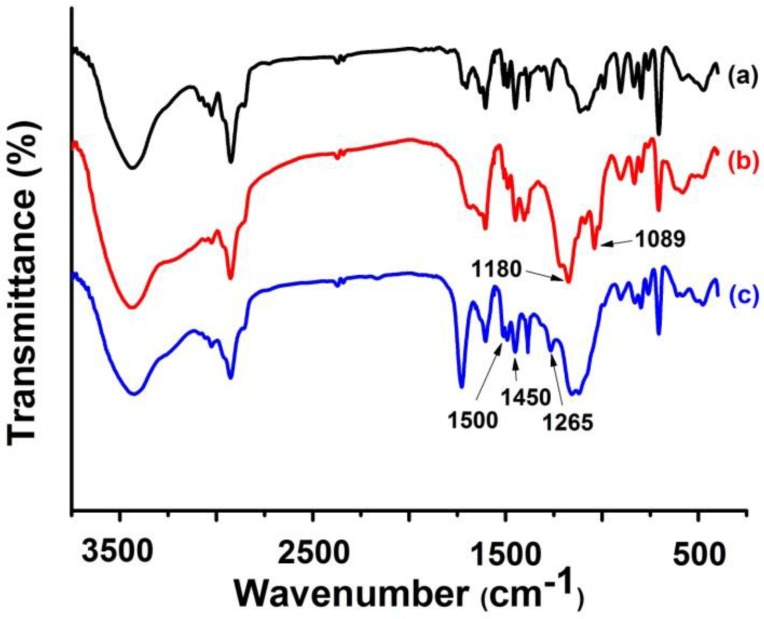
Fourier Transform Intrared(FT-IR) spectra of porous P(S-DVB) microparticles (**a**); sulfonated P(S-DVB) microparticles (**b**); and DR-modified P(S-DVB) microparticles (**c**).

As shown in Figure 8, the characteristic absorption peaks of sulfonic acid groups at 1180 and 1089 cm^−1^ indicate the successful sulfonation of porous P(S-DVB) microparticles. The absorption peak at 1265 cm^−1^ can be assigned to the stretching vibration of N-H derived from DR; the peaks at 1450 cm^−1^ and 1500 cm^−1^ were assigned to the stretching vibration of the aromatic ring of DR, demonstrating the presence of DR after the modification.

### 2.4. Chromatographic Performance

**Figure 9:**
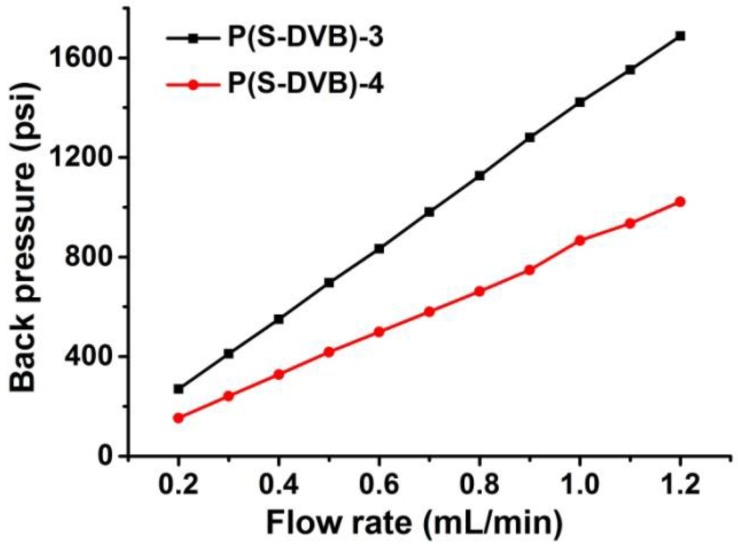
Back pressure of columns packed with different porous polymer microparticles: column, 75 mm × 4.6 mm Inner Diameter(I.D.); mobile phase, ACN–water mixture (*v*/*v* = 7:3).

To investigate their chromatographic performance, the porous P(S-DVB) microparticles were packed into stainless-steel chromatographic columns. Figure 9 shows the back pressure of columns packed with P(S-DVB)-3 and P(S-DVB)-4 at different flow rates. The back pressure demonstrates a decent linearity with the stream flow rate in the acetonitrile (ACN)–water mixture (*v*/*v* = 7:3). The back pressure of the column packed with P(S-DVB)-4 is lower than that of P(S-DVB)-3, because the average pore size of P(S-DVB)-4 (~18 nm) is bigger than that of P(S-DVB)-3 (~10 nm).

**Figure 10:**
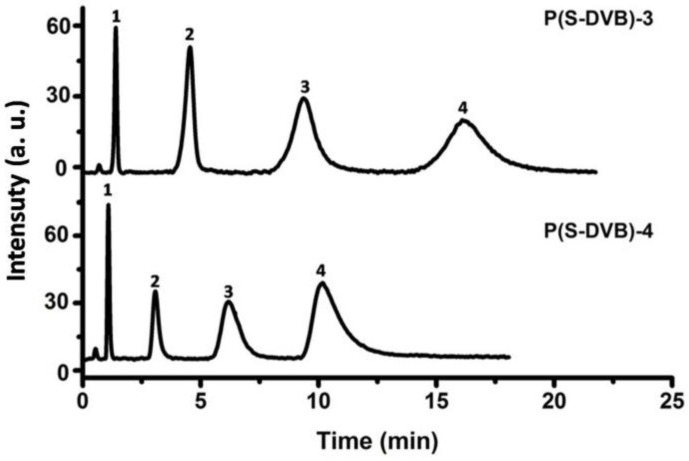
Chromatograms for the separation of benzene analogues on the porous P(S-DVB) column: column, 75 mm × 4.6 mm I.D.; injection size, 3 µL; flow rate: 0.5 mL/min; mobile phase, ACN–water mixture (*v*/*v* = 7:3); elution order: (1) uracil, (2) toluene, (3) naphthalene, (4) fluorene.

The mixture of uracil, toluene, naphthalene and fluorene were separated by the packed column with various permeable polymer microparticles. As shown in Figure 10, the column packed with P(S-DVB)-4 isolates the mixture faster than the column packed with P(S-DVB)-3 due to the higher specific surface area of the former. Because the P(S-DVB)-4 microparticle performed better than the P(S-DVB)-3 microparticle, it was selected for the DR surface modification and the following chromatographic tests.

**Figure 11:**
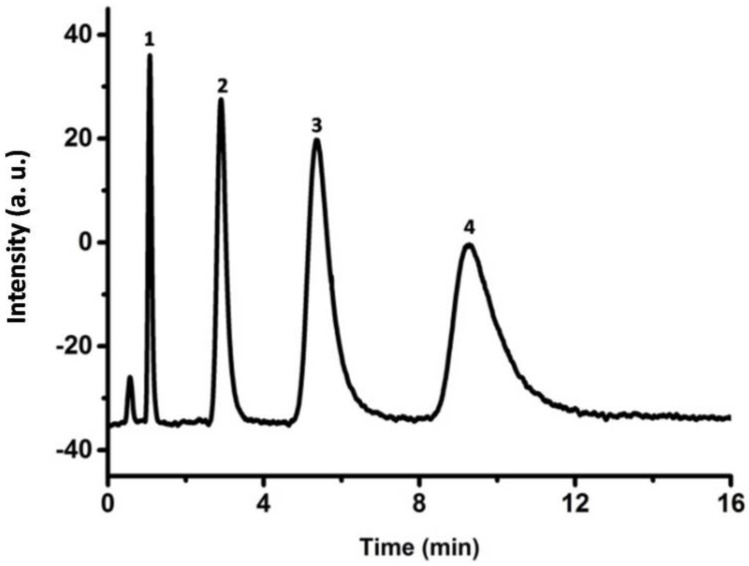
Chromatograms for the separation of benzene analogues on the porous P(S-DVB)-DR column: column, 75 mm × 4.6 mm I.D.; injection size, 3 µL; flow rate: 1.0 mL/min; mobile phase, ACN–water mixture (*v*/*v* = 7:3); elution order: (1) uracil, (2) toluene, (3) naphthalene, (4) fluorene.

Depending on the chemical structure of DR and mobile phase composition, the DR-modified porous P(S-DVB)-4 stationary phase performed different separation mechanisms, including reversed phase (RP) and hydrophilic interactions. As shown in Figure 11, the benzene analogues were also successfully separated by porous P(S-DVB)-DR microparticles. The affinity interaction between benzene rings of DR and the benzene analogues make the separation practical. Compared with Figure S2, the separation performance of the P(S-DVB)-DR column is better than a commercial C18 column (Agela Technologies, S185006, Wilmington, DE, USA) at the same 75 mm column length.

Table 2 shows that the run-to-run (*n* = 5) relative standard deviation(RSD) of retention time for the benzene analogues was less than 1%, day-to-day (*n* = 7) RSD was less than 2.5%, and column-to-column (*n* = 5) RSD was less than 3.5%. After a continuous 100 times running in one column, the RSDs of retention time for the benzene analogues were all less than 2.5%, and the separation performance of the P(S-DVB)-DR column was not deteriorated. Therefore, the P(S-DVB)-DR columns demonstrated very good stability and repeatability.

**Figure 12:**
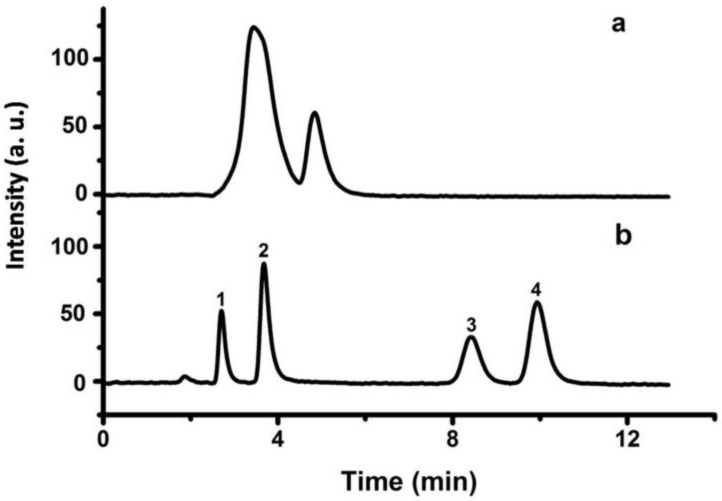
Chromatograms for the separation of four organic acids on the P(S-DVB)-4 column (**a**) and P(S-DVB)-DR (**b**) column: column, 75 mm × 4.6 mm I.D.; flow rate, 0.5 mL/min; injection size, 1 mL; mobile phase, phosphate buffer (pH = 4) and methanol (*v*/*v* = 85:15); elution order: (1) formic acid, (2) acetic acid, (3) lactic acid, (4) benzoic acid.

With the introduction of hydrophilic secondary amine groups on the surface of the P(S-DVB)-4 particles, a series of organic acids were baseline separated on the P(S-DVB)-DR column (Figure 12b). The bare P(S-DVB)-4 column (Figure 12a) was used to compare with the P(S-DVB)-DR column. As shown in Figure 12b, the P(S-DVB)-DR column could effectively separate four kinds of organic acids. However, for the bare P(S-DVB)-4 column, the resolution decreased significantly (Figure 12a). The chemical structure of DR on the surface of P(S-DVB)-4 particles enhanced the interactions between organic acids and the stationary phase so that the separation was much more efficient than bare P(S-DVB)-4 particles. Compared with Figure S3, the separation performance of the P(S-DVB)-DR column is better than a commercial C18 column (Agela Technologies, S185006) at the same 75 mm column length.

Table 3 shows that the run-to-run (*n* = 5) RSD of retention time for the organic acids was less than 1%, day-to-day (*n* = 7) RSD was less than 2%, and column-to-column (*n* = 5) RSD was less than 3.5%. After a continuous 100 times running in one column, the RSDs of retention time for the organic acids were all less than 2.5%, and the separation performance of the P(S-DVB)-DR column was not deteriorated. Thereby, the DR-modified P(S-DVB)-4 particles were robust and had excellent performance in mix-mode HPLC separations.

**Figure 13:**
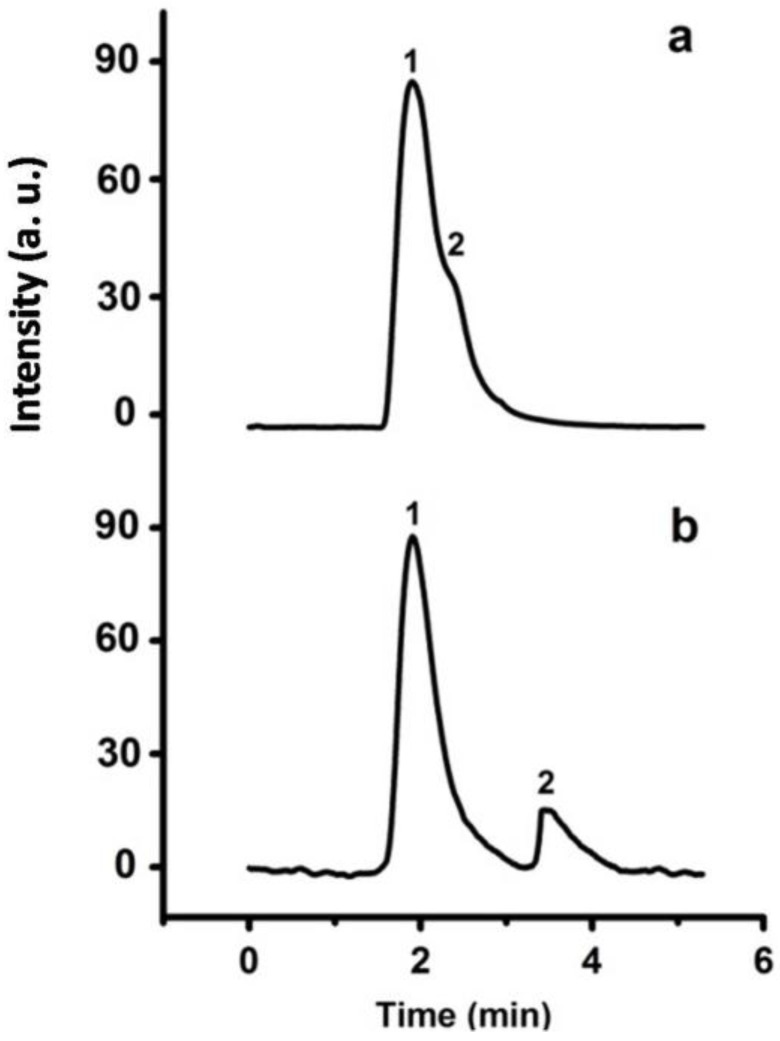
Chromatograms for the separation of C_60_ and C_70_ on the porous P(S-DVB)-4 column (**a**) and P(S-DVB)-DR (**b**) column: column, 75 mm × 4.6 mm I.D.; injection size, 3 µL; flow rate: 0.5 mL/min; mobile phase, hexane and isopropanol (*v*/*v* = 1:3.5); elution order: (1) C_60_, (2) C_70_.

According to the excellent performance of the P(S-DVB)-DR column in the separation of benzene analogues, C_60_ and C_70_—a class of special nanomaterials—were also separated in the P(S-DVB)-DR column using hexane and isopropanol (*v*/*v* = 1:3.5) as eluent. Considering that both C_60_ and C_70_ have carbon ring structures, the π–π affinity interactions between benzene rings of P(S-DVB)-DR particles and the carbon rings of fullerenes make the separation practical. As shown in Figure 13, C_60_ and C_70_ were successfully separated on the P(S-DVB)-DR column (Figure 13b), while they could not be separated on the bare P(S-DVB)-4 column (Figure 13a). Therefore, the DR surface modification contributed to the high efficiency of the P(S-DVB)-4 stationary phase for mix-mode separation. In the columns, C_60_—having less π–π bondings than C_70_—was first eluted due to the weaker interaction with the benzene rings of DR. Compared with Figure S4, the separation performance of the P(S-DVB)-DR column is better than a commercial C18 column (Agela Technologies, S185006) at the same 75 mm column length.

## 3. Materials and Methods 

### 3.1. Materials

Styrene (St, 99%), DVB (99%), AIBN (98%), sodium dodecylsulfate (SDS, 99%), DBP (99%), toluene (99.5%), naphthalene (99.5%), ethanol (99.5%), ACN (99.9%), isopropanol (99.7%) and THF (99%), copper chloride (CuCl_2_, 99%) were purchased from Tianjin Chemical company. Poly(*N*-vinylpyrrolidone) (PVP, M_n_ = 40,000) and polyvinyl alcohol (PVA-124, degree of polymerization 2400, degree of hydrolysis 98%) were provided by Sinopharm Chemical Reagent Company (Beijing, China). Uracil (99%) and fluorene (99.5%) were purchased from Aladdin Chemical Reagent Company (Shanghai, China). Benzoyl peroxide (BPO, 95%) was bought from Tianjin Beichen Chemical Company (Tianjin, China). DVB and St were used after vacuum distillation. DR (M_n_ = 2500) was synthesized by polycondensation of diphenylamine-4-diazonium salt and paraformaldehyde in concentrated H_2_SO_4_ according to a method described elsewhere [34].

### 3.2. Preparation of PS Seed Particles

Monodisperse PS seed particles with an average diameter of 2.7 µm were synthesized by a reported dispersion polymerization method [35]. The polymerization was carried out in a three-necked flask with mechanical stirring at 300 rpm under nitrogen atmosphere. In the flask, 1.35 g of PVP was dissolved in 80 g of ethanol. An amount of 15 g of styrene and 0.44 g of AIBN were also added into the flask after increasing the bath temperature to 70 °C. After 24 h of polymerization, the PS seed particles were separated through centrifugation and washed with ethanol three times, and then dried under vacuum at ambient temperature.

### 3.3. Synthesis of Porous Polymer Microparticles

Porous polymer particles were synthesized by two-step seeded swelling polymerization techniques as given below: seed particles (2.7 μm, 0.26 g) were ultra-sonicated to be well dispersed in 10 mL distilled water. A mixture of 1.2 mL toluene and 1.2 mL DBP was emulsified in 20 mL 0.375 wt % SDS aqueous solution in an ultrasonic bath (10 min, 100 W) separately. The dispersion of PS particles and emulsion of porogens were mixed and mechanically stirred at 300 rpm in an oil-bath at 35 °C for 24 h. A mixture of 0.6 mL styrene and 2.0 mL DVB containing 0.12 g BPO was emulsified in 30 mL 0.25% SDS aqueous solution using ultrasonic agitation (10 min, 100 W). The prepared emulsion was added into the swelled PS dispersion solution and stirred for another 24 h. At the end, 3.5 mL of 10% PVA aqueous solution was added into the dispersion. At the polymerization stage, the dispersion was purged with nitrogen for 30 min. Polymerization of the monomer phase in the swollen seed particles was carried out at 70 °C under mechanical stirring of 120 rpm for 24 h. After polymerization, the particles were isolated, washed with ethanol three times by using a centrifugation–decantation protocol, and then extracted with THF at 60 °C for 12 h to remove linear polymer and swelling agent completely. After centrifugation, the porous microparticles were washed with ethanol and dried in a vacuum oven.

### 3.4. Preparation of DR-Modified Porous P(S-DVB) Microparticles

Modification of porous P(S-DVB) microspheres with DR was carried out as follows: the porous P(S-DVB) microparticles (0.5 g) were first sulfonated by concentrated sulfuric acid (20 mL) at 40 °C for 4 h. The sulfonated porous P(S-DVB) microspheres were then added into 10 mL aqueous solution of DR (4 mg/mL) under magnetic stirring. The modified porous microspheres were washed with deionized water three times by centrifugation, and this process was repeated another three times in order to ensure that DR was completely coated onto the porous microspheres. After being dried under vacuum at room temperature for 12 h, the DR-coated P(S-DVB) microspheres were exposed under 365 nm UV light with an intensity of 350 mW/cm^2^ for 15 min. Consequently, DR was covalently linked to the surface of porous P(S-DVB) microspheres [36], and the yield was 95%.

### 3.5. Characterization

The surface morphology and structure of PS seed particles and porous P(S-DVB) spheres were observed by using scanning electron microscopy (SEM, JEOL JSM-6309LV, Beijing, China) and transmission electron microscopy (TEM, JEOL JEM-1200). An accelerated surface area and porosimetry analyzer (ASAP, Micromeritics 2020, Micromeritics Instrument Corp., Shanghai, China) was used for the BET tests. Chromatographic study was carried out using a HPLC (SEV P500, Qingdao Qicai Sunshine Information Technology Co., Ltd., Qingdao, China) equipped with a UV detector.

### 3.6. Chromatography

The monodisperse porous PS microparticles were dispersed in isopropanol/methanol (*v*/*v* = 1:1) and packed into stainless-steel columns (75 mm × 4.6 mm, I.D.) by applying a chromatographic column packing machine (GLK 2000, GALAK, Shanghai Yuhong Energy Saving Technology Co., Ltd., Shanghai, China) under a pressure of 18 MPa. The column was connected to the HPLC framework and flushed with methanol at a flow rate of 0.8 mL/min for 2 h to equilibrate the column until a constant UV baseline was obtained. A mixture of uracil, toluene, naphthalene and fluorene was separated in the HPLC column packed with porous P(S-DVB)-4 and P(S-DVB)-DR microparticles at a flow rate of 0.5‒1.0 mL/min at room temperature applying a UV detecting wavelength of 254 nm with ACN–water mixture (*v*/*v* = 7:3) as eluent. The organic acids mixture of formic acid, acetic acid, lactic acid and benzoic acid was also separated on the above two kinds of column under a flow rate of 0.5 mL/min at room temperature with phosphate buffer (pH = 4) and methanol (*v*/*v* = 85:15) as mobile phase at a detecting wavelength of 210 nm. In addition, C_60_ and C_70_ were separated on the modified P(S-DVB)-DR column using hexane and isopropanol (*v*/*v* = 1:3.5) as the mobile phase at a flow rate of 0.5 mL/min at a detecting wavelength of 290 nm.

## 4. Conclusions

In this paper, porous P(S-DVB) microparticles with high specific surface areas were successfully synthesized by the two-step activated swelling method. The effects of the porogens, swelling temperature and crosslinking agent were investigated in detail. The largest surface area is obtained when the toluene to DBP porogens ratio is 3:1, and porous P(S-DVB) microparticles have the roughest surface when a swelling temperature of 25 °C is applied. The obtained P(S-DVB) particles were modified by sulfonation and self-assembly positive charged photosensitive DR on the surface. After treatment with UV light, the ionic bonding between sulfonated P(S-DVB) and DR was converted into covalent bonding through a unique photochemistry reaction of DR. Depending on the chemical structure of DR and mobile phase composition, the P(S-DVB)-DR stationary phase performed different separation mechanisms, including reversed phase and hydrophilic interactions. Thus, a variety of baseline separations of benzene analogues and organic acids was achieved by using the P(S-DVB)-DR particles as packing materials in HPLC. According to the π–π interactional difference between carbon rings of fullerenes and benzene rings of P(S-DVB)-DR, C_60_ and C_70_ were also successfully separated in the HPLC column packed with P(S-DVB)-DR particles. Compared with bare P(S-DVB) packing material, the DR surface modification contributed to the high efficiency of the P(S-DVB) stationary phase for mix-mode separation.

## Figures and Tables

**Table 1:** Preparation conditions of porous polymer microparticles.

Sample	Toluene (mL)	DBP (mL)	Swelling Temperature (°C)	DVB (mL)	EDMA (mL)
P(S-DVB)-1	0	2.4	35	2.0	0
P(S-DVB)-2	0.6	1.8	35	2.0	0
P(S-DVB)-3	1.2	1.2	35	2.0	0
P(S-DVB)-4	1.8	0.6	35	2.0	0
P(S-DVB)-5	2.4	0	35	2.0	0
P(S-DVB)-6	1.2	1.2	15	2.0	0
P(S-DVB)-7	1.2	1.2	25	2.0	0
P(S-DVB)-8	1.2	1.2	45	2.0	0
P(S-EDMA)	1.2	1.2	35	0	2.0

^a^ Dibutyl phthalate(DBP); divinylbenzene(DVB); ethylene dimethacrylate(EDMA).

**Table 2:** Separation performance of the P(S-DVB)-DR column for benzene analogues.

Retention Time RSD (%)				
Benzene Analogues ^a^	Run to Run	Day to Day	Column to Column	Continuous 100 Times Running
	(*n* = 5)	(*n* = 7)	(*n* = 5)	
Uracil	0.56	1.54	2.21	1.65
Toluene	0.79	1.65	2.89	1.86
Naphthalene	0.81	1.87	3.32	2.16
Fluorene	0.86	2.23	3.47	2.32

^a^ Separation conditions: the same as Figure 11.

**Table 3:** Separation performance of the P(S-DVB)-DR column for organic acids.

Retention Time RSD (%)				
Organic Acids ^a^	Run to Run	Day to Day	Column to Column	Continuous 100 Times Running
	(*n* = 5)	(*n* = 7)	(*n* = 5)	
Formic Acid	0.81	1.84	2.46	2.12
Acetic Acid	0.75	1.65	2.32	1.93
Lactic Acid	0.98	1.98	3.43	2.43
Benzoic Acid	0.92	1.89	3.12	2.24

^a^ Separation conditions: the same as Figure 12.

## Supplementary Materials

The following are available online at www.mdpi.com/1996-1944/10/4/440/s1, Figure S1: Nitrogen adsorption-desorption isotherms of P(S-DVB)-DR. Inset is the distributions of pore size. Figure S2: Separation on C18 column for benzene analogues. Column, 75 mm × 4.6 mm I.D.; injection size, 3 µL; mobile phase, methanol.
