# Supplementary material for: Preparation of Porous Poly(Styrene-Divinylbenzene) Microspheres and Their Modification with Diazoresin for Mix-Mode HPLC Separations
Source: Materials (Basel). 2017 Apr 22;10(4):440. doi: 10.3390/ma10040440 (PMC5506891; doi:10.3390/ma10040440)
Supplement: Supplementary file 1 [file materials-10-00440-s001.pdf]

Supplementary Materials: Preparation of Porous Poly(Styrene-Divinylbenzene) Microspheres and Their Modification with Diazoresin for Mix-Mode HPLC Separations

Bing Yu, Tao Xu, Hailin Cong, Qiaohong Peng and Muhammad Usman

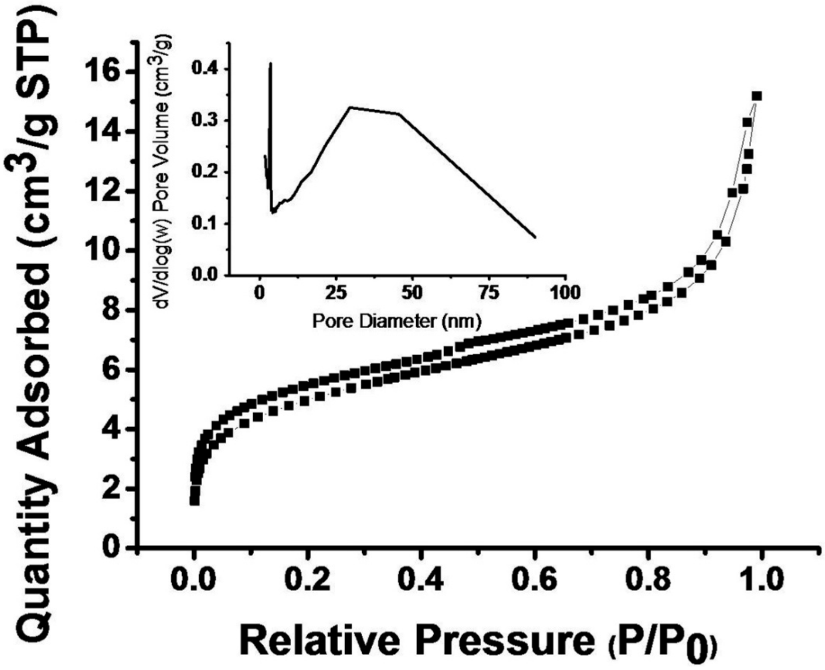

Figure S1. Nitrogen adsorption-desorption isotherms of P(S-DVB)-DR. Inset is the distributions of pore size.

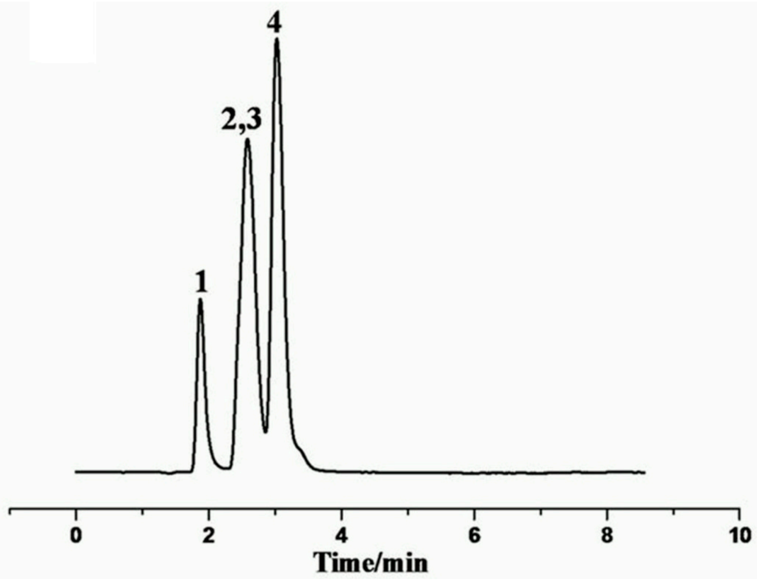

Figure S2. Separation on C18 column for benzene analogues. Column, 75 mm × 4.6 mm I.D.; injection size, 3 μL; mobile phase, methanol.

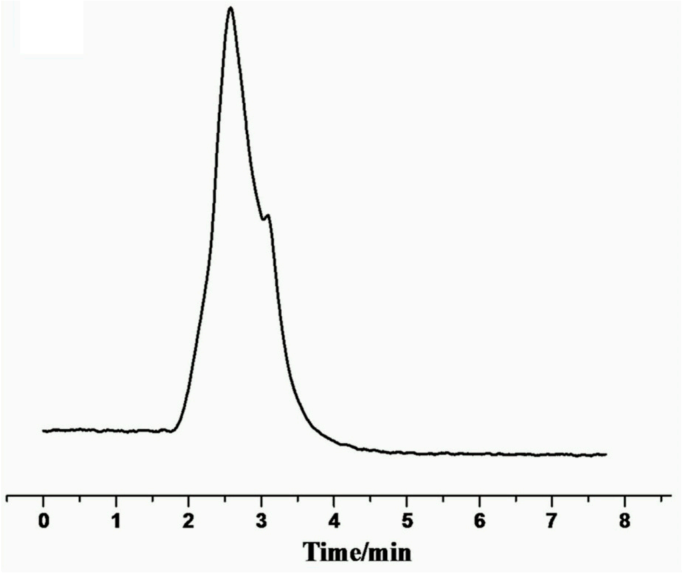

Figure S3. Separation on C18 column for organic acids. Column, 75 mm × 4.6 mm I.D.; flow rate, 0.5 ml/min; injection size, 3 μL; mobile phase, phosphate buffer (pH = 4) and methanol (*v*/*v* = 85:15).

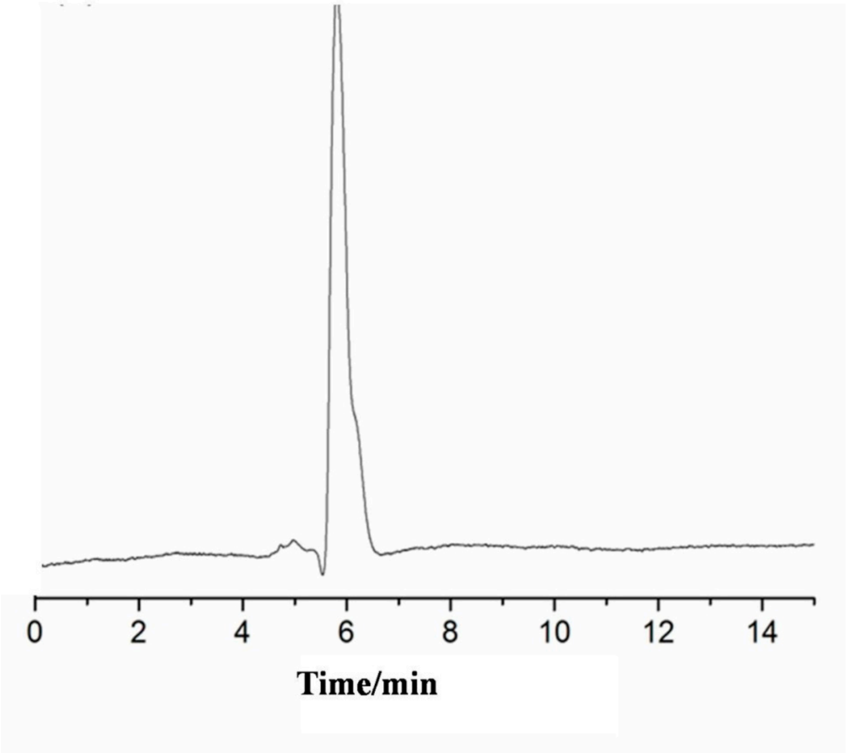

Figure S4. Separation on C18 column for C60 and C70: column, 75 mm × 4.6 mm I.D.; injection size, 2 μL; flow rate: 0.15 mL/min; mobile phase, hexane and isopropanol (*v*/*v* = 1:3).

© 2017 by the authors. Submitted for possible open access publication under the terms and conditions of the Creative Commons Attribution (CC-BY) license (http://creativecommons.org/licenses/by/4.0/).
